# Mr. Ryal, on Cold Affusion in Colic

**Published:** 1805-01-01

**Authors:** J. Ryal

**Affiliations:** late Surgeon of the Royal Navy. Braunton


### *To the Editors of the Medical and Physical Journal.*

Gentlemen,

A Remedy has been proposed in the Medical Essays, for the relief of patients labouring under spasmodic colic, attended with obstinate costiveness; and although some advantage has been ascribed to it, a single case never came within my knowledge of its having been tried, before that which I am now going to relate. The remedy I here allude to is, after having got the patient out of bed, to make him walk on a cold damp floor, and to throw cold water on his feet, legs, and thighs.

Several months ago I was called to see Mrs. Lock, ætat. thirty, of healthy habit; she complained of a violent pain in the umbilical region, extending downwards; described the sensation of a twisting and burning, and had not been at at stool for more than thirty hours. Her disorder she attributed to a bit of ox-tripe which she had eaten a few days before, and which, she said, she was sure she had not digested. Her pulse was frequent, and a considerable degree of pyrexia with violent retching had come on. I immediately gave her twenty grains of ipecac. with a view to remove the indigestible substance; but this, instead of affording any relief, increased the existing symptoms. I then gave her the mixtur. salin. with a few drops of the tincture opii in each dose; an emollient clyster was given every three hours, and the abdomen fomented. The clysters were returned unchanged, and gave no ease. The pyrexia was now in a great measure diminished, and I tried alternately strong purgatives and more stimulant injections, but with no greater success. The cathartics were almost immediately rejected, and the clysters were returned without producing the least discharge of fæces, leaving behind the most exquisite torture. I ordered the patient to sit over the steam of warm water, applied a blister to the abdomen, (though from the latter I did not expect much benefit) and gave her small doses of the ol. Ricin. combined with the aq. menth. every half hour, adding to each dose ten drops of the tinct. opii. The dose of the tinct. opii was afterwards increased, before I could procure the least respite of ease, from the violent irritation in her stomach and bowels, which had been produced by the use of the cathartics.

I frequently found in this complaint, that purgatives of every kind had failed to produce stools, until large doses of the laudanum had been first administered; but here it only gave temporary relief, and the subsequent use of purges was alike ineffectual. Thus, after having had recourse in vain to all the methods I could devise to produce a stool, did my wretched patient drag on eight days and nights. In the mean time, I should gladly have availed myself of the advice of Dr. Wavell, physician, at Barnstaple, a gentleman whose experience, literary and professional knowledge, and urbanity of manners, peculiarly calculate him for the importance of his situation, but business of an urgent nature had called him to London. I recommended some of the neighbouring surgeons or apothecaries to be called in, but neither my patient nor her friends would listen to it. This confidence in me, who had been only a very short time in practice in this place, instead of gratifying, made me the more unhappy.

The ninth morning after my patient had been seized, had now arrived, and brought with it appearances of a very distressing and alarming nature; her countenance was now very much emaciated, her breath fœtid, pulse small and intermittent; had convulsive hiccoughs, and clammy sweats. The violence of the pain still continued, but the fæces were not vomited, and these were the only circumstances that were not very unfavourable. I had heard much of the benefit derived from swallowing leaden bullets and quick-silver; but so strong was my prejudice against this practice, that I could not prevail on myself to make trial of it, and determined on the application of cold to the lower extremities. This was to me an experiment attended with much hazard, for had not my patient recovered, after undergoing an operation never before heard of in this place, the sapient sneer and significant shake of the head of some of my medical brethren, together with the vulgar prejudice, would have ascribed her death to my temerity, and passed judgment upon me.

After having with much difficulty, though assisted by two women, got the patient out of bed, I supported her to the next apartment, and made her walk barefoot over the floor, which had been previously spread over with table cloths just taken out of a tub of cold water; I also dashed cups of that fluid with some violence against her feet, legs, and thighs; within five minutes, instead of becoming weak by being taken out of bed, she felt stronger, and able to move with less support, and her strength kept gradually increasing; in fifteen minutes a severe griping pain came on, which continued for about ten minutes longer, when she was happily relieved by a very copious discharge of fetid discoloured fæces.

Though very much weakened from this discharge, she said she fancied herself in heaven; but this by no means removed my fears. The extreme debility which came on, her sudden relief from pain, the fetor and colour of the discharge, joined to the state of her pulse, made me suspect all was not right. I got her into bed, ordered her some sago, which, with the exception of a little gruel, was the only food she had taken from the commencement of her complaint; to the sago was added some port with the tinct. opii. The cort. cinch. combined with the rad. rhei, was given, and I found my fears were groundless; for she recovered in a very short time.

Before I would take the liberty of requesting a place for this case in your no less amusing than instructing publication, tion, I determined to give the method some more trials. In this part of Devonshire, cyder is particularly abundant, and consequently the Devonshire colic is no rare occurrence. Whenever habitual costiveness attended this disease I used the application of cold to the feet, and it produced the desired effect; before I was convinced of its efficacy I should have expected more benefit from the warm pediluvium, but now the former has my decided preference.

The stimulus of cold applied to the feet is conveyed to the intestines, and produces downwards their peristaltic motion; this I conceive to be the modus operandi. It is well known that the unusual exposure of the feet to wet or cold often produces griping and diarrhœa; the erect posture also and motion which the patient uses, no doubt, assist in the operation of the remedy.

*Braunton,* I am, &c.

*Nov.* 9, 1804. J. RYAL, late Surgeon of the Royal Navy.

